# The Impact of the COVID‐19 Pandemic on Cardiac Related Emergency Department Presentations in Queensland: A Retrospective Cohort Study

**DOI:** 10.1111/1742-6723.70096

**Published:** 2025-07-24

**Authors:** Emma J. Hall, Gerben Keijzers, Jamie Ranse, Amy L. Sweeny, Julia Crilly, COVERED‐COVID Study Investigators, Julia Crilly, Amy Sweeny, Gerben Keijzers, John Gerrard, Dinesh Palipana, Jamie Ranse, Jaimi Greenslade, Ben Walters, Anthony Padowitz, David Green, Andrea Marshall, Ping Zhang, Gary Grant, Yang D. Teng, Ben Gerhardy, Kylie Alcorn, Emma Hall

**Affiliations:** ^1^ School of Pharmacy and Medical Science Griffith University Gold Coast Australia; ^2^ Department of Emergency Medicine Gold Coast Hospital and Health Service Southport Australia; ^3^ Faculty of Health Sciences and Medicine Bond University Robina Australia; ^4^ School of Medicine and Dentistry Griffith University Gold Coast Australia; ^5^ School of Nursing and Midwifery Griffith University Gold Coast Australia

**Keywords:** chest pain, COVID‐19 pandemic, emergency service, heart disease, hospital

## Abstract

**Objective:**

To (i) describe and compare rates, demographics, ED characteristics and outcomes for cardiac‐related presentations to Queensland EDs before, during and after periods of government restrictions, (ii) determine if and which cardiac conditions were impacted by COVID‐19 restrictions.

**Methods:**

Retrospective cohort study of all adult presentations in 105 Queensland public EDs who were diagnosed with a cardiac condition or chest pain. Four periods were compared: ‘pre‐pandemic’ (January 2018–March 2020), ‘statewide restrictions’ (11 March–30 June 2020), ‘easing of restrictions’ (1 July 2020–12 December 2021) and ‘outbreak’ (13 December 2021–30 June 2022). ED presentation rates (per 10,000 person‐years) and incident rate ratios were calculated for chest pain, ischaemic heart disease, arrhythmias, heart failure, inflammatory conditions, cardiac arrest, and ‘other’ acute cardiac conditions. Proportions of presentations by demographic group, ED characteristics and outcomes were also compared.

**Results:**

The study included 609,485 acute cardiac‐related presentations. All‐cause acute cardiac presentations decreased by 4% from ‘pre‐pandemic’ to ‘statewide restrictions’, then increased by 20% and 25% in the ‘easing of restrictions’ and ‘outbreak’ periods, respectively. Ischaemic heart disease presentation rates decreased during ‘statewide restrictions’. Weekly chest pain presentations dropped early during ‘statewide restrictions’ but increased in each consecutive period. Weekly heart failure presentations appeared to follow seasonal patterns. Compared to ‘statewide restrictions’, inflammatory presentations increased during ‘easing of restrictions’ and ‘outbreak’ periods.

**Conclusions:**

The COVID‐19 pandemic impacted acute cardiac‐related ED presentations in Queensland in various ways. Public health messaging for people to seek timely medical care for urgent conditions and symptoms should be emphasised in future pandemics.

## 1 Introduction

The COVID‐19 pandemic, declared by the World Health Organization in March 2020, has reshaped global healthcare. EDs were considerably impacted, with reports from several countries showing reduced presentation rates. All‐cause ED presentations decreased between 31% and 66% in the United States and Italy in March and April 2020 [1, 2]. Australia observed a similar trend with up to 25% reductions in ED presentations during the first half of 2020 [3, 4, 5]. For cardiac‐related conditions, studies report varying changes in ED presentations, ranging from decreases of 12% to 76% in Italy [6], United States [7], and France [8], while in other locations (Turkey, Austria), presentation rates were unchanged [9, 10]. Additionally, there was an increase in out‐of‐hospital cardiac arrests with decreased survival rates in Europe and United States [11], two areas with high COVID‐19 case numbers, suggesting that changes in emergency care access may have affected patient outcomes. The majority of these studies focus on the first 12 months of the pandemic, with ongoing impacts less frequently reported.

In Queensland, COVID‐19 restrictions were initiated early, resulting in low case numbers. Following the vaccination of most adults in late 2021, Queensland reopened interstate and international borders in December 2021. The first two waves of COVID‐19 in Queensland commenced on 13 December 2021 and 14 March 2022, each lasting around 12 weeks [12]. While early and stringent lockdowns in Queensland minimised community transmission of the SARS‐CoV‐2 virus, the impact on cardiac‐related ED presentations and outcomes remains unclear.

With many acute cardiac conditions requiring urgent medical assessment and management, it is important to establish whether pandemic restrictions influenced this specific patient group. The aims of this study were to: (i) describe and compare the rates, patient demographics, ED characteristics and outcomes for acute cardiac‐related presentations made to Queensland EDs before, during and after periods of government restrictions, and (ii) to determine if and which specific cardiac conditions were impacted by the COVID‐19 restrictions.

## 2 Methods

### 2.1 Study Design

This was a statewide retrospective cohort study of all adult acute cardiac‐related presentations made to 105 Queensland public hospital EDs between 1 January 2018 and 30 June 2022.

### 2.2 Setting

The study was set in Queensland, a state with a population of 5.3 million in 2022 [13]. Queensland EDs assessed and diagnosed approximately 66,000 cardiovascular‐related patient presentations and managed an additional 82,000 presentations for ‘pain in the throat and chest’ in 2022–2023 [14].

### 2.3 Sample

The study sample comprised all adult (18 years and older) ED presentations that received a primary International Statistical Classification of Diseases, tenth revision, Australian modification (ICD‐10‐AM) diagnostic code relating to an acute cardiac condition or unspecified chest pain. The selection of 114 clinically meaningful ICD‐10‐AM codes used for this study were based on government reports [14, 15, 16], previous literature [7, 8], and discussion between the authors who have ED and cardiology experience. The ICD‐10‐AM codes were categorised into seven major cardiac condition groups (chest pain, ischaemic heart disease, arrhythmias, heart failure, inflammatory conditions, cardiac arrest, and ‘other’ acute cardiac conditions) that reflect pathological homogeneity [17]. The diagnoses Bradycardia, Tachycardia and Palpitations were considered to be signs/symptoms and not necessarily reflective of true cardiac‐related diagnoses at the conclusion of the ED presentation. As such the corresponding ICD‐10‐AM codes were included in the ‘Other acute cardiac conditions’ group. A list of the ICD‐10‐AM codes is provided in Table S1.

### 2.4 Data Source and Collection

Data were derived from the Emergency Data Collection (EDC), a minimum dataset that comprises routinely collected administrative and health data from 105 public EDs within Queensland [18]. Variables used from the EDC to meet the aim of this research included patient demographics (i.e., age, sex, Indigenous status, residential postcode and suburb), ED characteristics (i.e., facility ID, facility Hospital and Health Service area, arrival transport mode, arrival date and time, Australasian Triage Scale (ATS) category, episode end status), and ED presentation outcomes (i.e., principal diagnosis, discharge disposition, discharge date and time).

### 2.5 Data Analysis

Data cleaning and coding was performed to align with Australian Institute of Health and Welfare (AIHW) and Australian Bureau of Statistics (ABS) classifications (Table S2). The residential postcode of patients presenting to ED was used to categorise presentations by Socio‐Economic Indexes for Areas (SEIFA) Index of Relative Socio‐Economic Disadvantage (IRSD) [19] and by remoteness [20].

ED presentations were analysed according to four periods of interest: [1] ‘pre‐pandemic’—the period prior to the pandemic declaration (1 January 2018–10 March 2020); [2] ‘statewide restrictions’—where border closures and government restrictions were implemented within Queensland (11 March 2020–30 June 2020), [3] ‘easing of restrictions’—where restrictions gradually eased (1 July 2020–12 December 2021) and [4] Queensland COVID‐19 ‘outbreak’—where restrictions and border closures were lifted and the first two waves of community transmission occurred in Queensland (13 December 2021–30 June 2022) [12].

Data were analysed using IBM SPSS Statistics for Windows v29.0 (IBM Corp: Armonk, NY). Patient demographics, ED presentation characteristics, and outcomes were described and compared using chi‐square or Mann–Whitney *U* test, with a *p*‐value of < 0.05 indicating a statistically significant difference between ‘statewide restrictions’ as the reference period and each of the three comparison periods. Subsequent pairwise z‐tests were used to compare specific groups within each comparison. To describe changes between time periods, incidence rate ratios (IRR) and 95% confidence intervals (CI) were calculated using OpenEpi (www.openepi.com). Presentation rates per 10,000 person‐years for the cardiac conditions were calculated by dividing the number of actual presentations by the Estimated Resident Population of Queensland as of June 2020 and the proportion of the year represented by each time period.

## 3 Results

### 3.1 Presentation Rates

Over the four‐and‐a‐half‐year study period, 609,485 acute cardiac‐related presentations were made to Queensland public hospital EDs (Table 1). During ‘statewide restrictions’, the average number of all‐cause acute cardiac presentations decreased by 14 presentations per day (4% decrease) compared to the ‘pre‐pandemic’ period (347 vs. 333 presentations per day). Compared to ‘statewide restrictions’, there was a 20% increase in presentations (65 more presentations per day) during ‘easing of restrictions’ and a 25% increase (83 more presentations per day) in the ‘outbreak’ period.

**TABLE 1:** Demographic characteristics of acute cardiac‐related ED presentations, by time period.

Characteristics	All visits (1642 days) (*n* = 609,485)		Pre‐pandemic period (800 days) (*n* = 277,877)			Statewide restrictions period (112 days) (*n* = 37,288)				Easing of restrictions period (530 days) (*n* = 211,088)				Queensland COVID‐19 outbreak (200 days) (*n* = 83,232)			
	*n*	%	*n*	%	Rate per day	*n*	%	*p* ^a^	Rate per day	*n*	%	*p* ^b^	Rate per day	*n*	%	*p* ^c^	Rate per day
Presentations per day (all acute cardiac)					347				333				398				416
Sex								0.236				0.136				0.001	
Male	305,352	50.1	140,510	50.6	176	18,733	50.2		167	105,166	49.8		198	40,943	49.2	*	205
Female	304,070	49.9	137,337	49.4	172	18,551	49.8		166	105,905	50.2		200	42,277	50.8	*	211
Other/Missing	63	0.0															
Age mean (SD)	56.6 (19.1)		57.7 (18.9)			57.2 (18.9)		< 0.001		56.1 (19.2)		< 0.001		54.4 (19.5)		< 0.001	
Age category (years)								< 0.001				< 0.001				< 0.001	
< 25	35,332	5.8	14,365	5.2	18	1955	5.2	*	17	13,071	6.2	*	25	5941	7.1	*	30
25–34	60,333	9.9	24,691	8.9	31	3463	9.3	*	31	21,992	10.4	*	41	10,187	12.2	*	51
35–44	74,226	12.2	32,095	11.6	40	4525	12.1		40	26,387	12.5	*	50	11,219	13.5	*	56
45–54	101,437	16.6	46,515	16.7	58	6170	16.5		55	34,940	16.6		66	13,812	16.6		69
55–64	108,592	17.8	50,182	18.1	63	6777	18.2		61	37,439	17.7	*	71	14,194	17.1	*	71
65–74	105,799	17.4	50,680	18.2	63	6700	18.0		60	35,672	16.9	*	67	12,747	15.3	*	64
75–84	83,079	13.6	39,399	14.2	49	5210	14.0		47	28,041	13.3	*	53	10,429	12.5	*	52
85+	40,651	6.7	19,932	7.2	25	2487	6.7	*	22	13,529	6.4		26	4703	5.7	*	24
Aboriginal or Torres Strait Islander origin								0.016				0.245				0.188	
Aboriginal, torres strait islander or both	47,311	7.8	20,733	7.5	26	2931	7.9	*	26	17,037	8.1		32	6610	8.0		33
Neither	559,414	92.2	255,770	92.5	320	34,186	92.1	*	305	193,159	91.9		364	76,299	92.0		381
Missing	2760	0.0															
Socioeconomic status (SEIFA)								0.695				0.116				< 0.001	
Most disadvantaged 1st quintile	160,853	26.6	74,396	27.0	93	9955	26.9		89	55,269	26.3		104	21,233	25.7	*	106
2nd quintile	143,471	23.7	65,331	23.7	82	8755	23.6		78	49,970	23.8		94	19,415	23.5		97
3rd quintile	132,955	22.0	60,355	21.9	75	8110	21.9		72	46,075	22.0		87	18,415	22.3		92
4th quintile	111,291	18.4	50,097	18.2	63	6853	18.5		61	38,878	18.5		73	15,463	18.7		77
Least disadvantaged 5th quintile	56,227	9.3	25,096	9.1	31	3351	9.1		30	19,657	9.4		37	8123	9.8	*	41
Missing (overseas/unknown)	4688																
Remoteness (residential address)								< 0.001				0.009				0.158	
Major cities of Australia	337,050	55.5	153,479	55.4	192	20,688	55.7		185	116,864	55.5		220	46,019	55.5		230
Inner regional Australia	147,563	24.3	67,353	24.3	84	8931	24.0		80	51,340	24.4		97	19,939	24.0		100
Outer regional Australia	100,785	16.6	45,685	16.5	57	6149	16.5		55	34,913	16.6		66	14,038	16.9		70
Remote Australia	9486	1.6	4279	1.5	5	615	1.7		5	3320	1.6		6	1272	1.5		6
Very remote Australia	9575	1.6	4422	1.6	6	613	1.6		5	3241	1.5		6	1299	1.6		6
Overseas	2492	0.4	1556	0.6	2	129	0.3	*	1	530	0.3	*	1	277	0.3		1
No fixed address	772	0.1	298	0.1	0	45	0.1		0	292	0.1		1	137	0.2		1
Missing	1762																
Residential location								< 0.001				< 0.001				< 0.001	
Interstate	8262	1.4	4674	1.7	6	203	0.5	*	2	2251	1.1	*	4	1134	1.4	*	6
Overseas	2498	0.4	1557	0.6	2	130	0.3	*	1	533	0.3	*	1	278	0.3		1
Queensland	596,736	98.2	270,712	97.8	338	36,834	99.1	*	329	207,661	98.7	*	392	81,529	98.3	*	408
Missing/unknown	1989		934			121				643				291			

Abbreviations: SD, standard deviation; SEIFA, socio‐economic indexes for areas.

* Indicates *p* < 0.05 on pairwise z‐test between the proportion of cases within each subcategory during the statewide restriction period and comparison period.

^a^ Compares pre‐pandemic versus statewide restrictions periods.

^b^ Compares statewide restrictions period versus easing of restrictions.

^c^ Compares statewide restrictions period versus QLD COVID‐19 outbreak period.

### 3.2 Patient Demographics

Sex distribution was relatively even among presentations (50.1% male) and did not change greatly over time (range 49.2% to 50.6%, Table 1). Over the study period, the mean age of patients decreased from 57.7 to 54.4 years. The proportion of presentations made by patients under the age of 45 years increased from 26.6% during ‘statewide restrictions’ to 32.8% during the ‘outbreak’ period. There was a small, albeit statistically significant increase (0.4%, *p* = 0.016) in presentations made by Aboriginal and/or Torres Strait Islander patients during ‘statewide restrictions’, which remained the same for the rest of the study period.

The proportion of presentations made by patients in different SEIFA categories did not change until the ‘outbreak’ period, where very minimal, but statistically significant, changes occurred, including a small decrease (−0.4%, *p* < 0.001) in presentations made by the most disadvantaged people and a small increase (0.7%, *p* < 0.001) in presentations made by the least disadvantaged people. There was no change in the proportions of patients presenting from major cities, regional or remote areas of Queensland. A significant decrease in the proportion of patients residing interstate and overseas was evident following ‘statewide restrictions’.

### 3.3 ED Presentation Characteristics

ED presentation characteristics and outcomes are summarised in Table 2. A small but statistically significant difference in the proportion of ambulance presentations, ATS category two allocations, and arrival time of day was noted during ‘statewide restrictions’. Small differences in ED presentation characteristics continued through the ‘easing of restrictions’ and ‘outbreak’ periods.

**TABLE 2:** Emergency department presentation characteristics and outcomes of acute cardiac‐related ED presentations, by time period.

Characteristics	All visits (1642 days) (*n* = 609,485)		Pre‐pandemic period (800 days) (*n* = 277,877)			Statewide restrictions period (112 days) (*n* = 37,288)				Easing of restrictions period (530 days) (*n* = 211,088)				Queensland COVID‐19 outbreak (200 days) (*n* = 83,232)			
	*n*	%	*n*	%	Rate per day	*n*	%	*p* ^a^	Rate per day	*n*	%	*p* ^b^	Rate per day	*n*	%	*p* ^c^	Rate per day
Presentations per day (all acute cardiac)					347				333				398				416
Day of arrival								0.941				0.458				0.243	
Weekday	460,414	75.5	210,177	75.6	—	28,210	75.7		—	159,319	75.5		—	62,708	75.3		—
Weekend	149,071	24.5	67,700	24.4	—	9078	24.3		—	51,769	24.5		—	20,524	24.7		—
Time of arrival								0.007				0.004				0.055	
In—hours: 06:00–17:59	390,921	64.1	178,778	64.3	224	23,726	63.6	*	212	135,937	64.4	*	257	52,480	63.1		262
Out of hours: 18:00–05:59	218,564	35.9	99,099	35.7	124	13,562	36.4	*	121	75,151	35.6	*	142	30,752	36.9		154
Mode of arrival								< 0.001				< 0.001				< 0.001	
Ambulance	321,321	52.7	149,360	53.8	187	20,507	55.0	*	183	110,230	52.2	*	208	41,224	49.5	*	206
Police	745	0.1	270	0.1	0	45	0.1		0	308	0.1		1	122	0.1		1
Walk—in	284,942	46.8	127,010	45.7	159	16,607	44.5	*	148	99,713	47.2	*	188	41,612	50.0	*	208
Other	2477	0.4	1237	0.4	2	129	0.3	*	1	837	0.4		2	274	0.3		1
Australasian triage scale category								0.001				< 0.001				< 0.001	
1	10,438	1.7	5066	1.8	6	624	1.7	*	6	3454	1.6		7	1294	1.6		7
2	392,250	64.4	174,321	62.7	218	23,732	63.6	*	212	140,248	66.4	*	265	53,949	64.8	*	270
3	178,004	29.2	85,163	30.6	107	11,214	30.1	*	100	57,953	27.5	*	109	23,674	28.4	*	118
4	24,852	4.1	11,967	4.3	15	1573	4.2		14	7969	3.8	*	15	3343	4.0		17
5	3941	0.6	1360	0.5	2	145	0.4	*	1	1464	0.7	*	3	972	1.2	*	5
End status								< 0.001				< 0.001				< 0.001	
Admit	393,929	60.0	192,728	69.4	241	24,468	65.6	*	219	133,069	63.0	*	251	43,664	52.5	*	218
Discharge	215,556	40.0	85,149	30.6	106	12,820	34.4	*	115	78,019	37.0	*	147	39,568	47.5	*	198

* Indicates *p* < 0.05 on pairwise z‐test between the proportion of cases within each subcategory during the statewide restriction period and comparison period.

^a^ Compares pre‐pandemic versus statewide restrictions periods.

^b^ Compares statewide restrictions period versus easing of restrictions.

^c^ Compares statewide restrictions period versus QLD COVID‐19 outbreak period.

### 3.4 ED Presentation Outcomes

During and after the ‘statewide restrictions’, the proportion of presentations resulting in hospital admission reduced from 65.6% during ‘statewide restrictions’ to 63.0% (*p* < 0.001) during ‘easing of restrictions’, and to 52.5% (*p* < 0.001) in the ‘outbreak’ period (Table 2). Overall ED length of stay was shorter during ‘statewide restrictions’ (−14 min) and ‘outbreak’ (−17 min) periods, compared to the ‘pre‐pandemic’ period (Table 3). The greatest reduction in length of stay (−34 min) was in people admitted during the ‘outbreak’ period (173 min) compared to ‘pre‐pandemic’ (207 min).

**TABLE 3:** Emergency department length of stay of acute cardiac‐related ED presentations, by time period.

Time outcomes	All visits (*n* = 609,485)		Pre‐pandemic period (*n* = 277,877)		Statewide restrictions period (*n* = 37,288)		Easing of restrictions period (*n* = 211,088)		Queensland COVID‐19 outbreak (*n* = 83,232)		*p*, Pre‐pandemic versus statewide restrictions	*p*, statewide restrictions versus easing	*p* srestrictions versus outbreak
ED length of stay minutes (median, IQR)	200 (129, 303)		204 (133, 305)		190 (124, 288)		200 (129, 308)		187 (117, 293)		< 0.001	< 0.001	< 0.001
ED length of stay minutes (admissions only; median, IQR)	198 (126, 310)		207 (134, 323)		188 (122, 291)		193 (124, 306)		173 (106, 280)		< 0.001	< 0.001	< 0.001
ED length of stay minutes (discharges only; median, IQR)	203 (133, 294)		197 (131, 277)		193 (128, 283)		211 (138, 309)		202 (130, 302)		0.095	< 0.001	< 0.001
*Outcome*	*n*	%	*n*	%	*n*	%	*n*	%	*n*	%	*p*	*p*	*p*
Admissions with ED LOS < 4 h	247,490	63.3	117,311	61.6	16,172	66.4	84,268	63.7	29,739	68.2	< 0.001	< 0.001	< 0.001
Admissions with ED LOS ≥ 4 h	143,181	36.7	73,212	38.4	8180	33.6	47,932	36.3	13,857	31.8	< 0.001	< 0.001	< 0.001
Discharges with ED LOS < 4 h	135,366	62.8	56,868	66.8	8444	65.9	46,066	59.1	23,988	60.7	0.034	< 0.001	< 0.001
Discharges with ED LOS ≥ 4 h	80,051	37.2	28,215	33.2	4371	34.1	31,905	40.9	15,560	39.3	0.034	< 0.001	< 0.001

Abbreviations: ED, emergency department; IQR, interquartile range; LOS, length of stay.

### 3.5 Acute Cardiac Conditions

**FIGURE 1:**
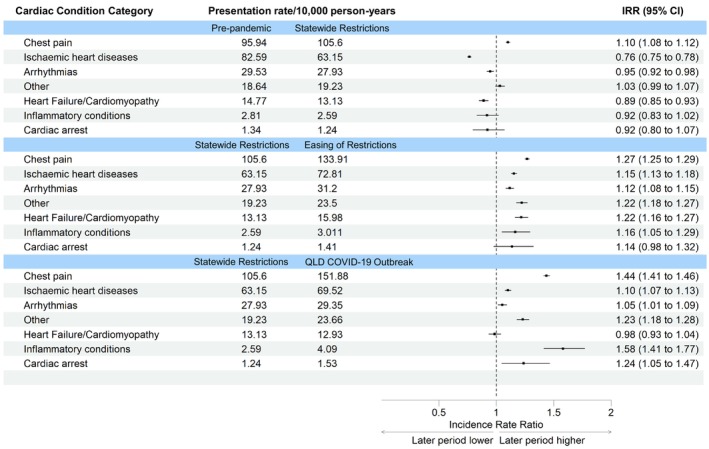
Presentation rates per 10,000 person‐years for acute cardiac conditions.

Compared to ‘pre‐pandemic’, there was a significant increase in presentations for chest pain during ‘statewide restrictions’ (IRR: 1.10, 95% CI: 1.08–1.12) (Figure 1). Ischaemic heart disease presentations decreased during ‘statewide restrictions’ (IRR: 0.76, 95% CI: 0.75–0.78), as did arrhythmias and heart failure presentations to a lesser extent. Presentations for cardiac arrest and other acute cardiac conditions did not change significantly.

Compared to ‘statewide restrictions’, chest pain presentations continued to increase in both ‘easing of restrictions’ (IRR: 1.27, 95% CI: 1.25–1.29) and ‘outbreak’ periods (IRR: 1.44, 95% CI: 1.41–1.46). Ischaemic heart disease presentations increased after ‘statewide restrictions’, but not to ‘pre‐pandemic’ levels. Arrhythmias decreased to ‘pre‐pandemic’ levels. Presentations for other cardiac conditions increased to a higher rate after ‘statewide restrictions’. Heart failure presentations fluctuated over the four time periods. Inflammatory conditions increased in the ‘easing of restrictions’ (IRR: 1.16, 95% CI: 1.05–1.29) and ‘outbreak’ periods (IRR: 1.58, 95% CI: 1.41–1.77). Presentations for cardiac arrest remained unchanged before increasing during the ‘outbreak’ period (IRR: 1.24, 95% CI: 1.05–1.47).

**FIGURE 2:**
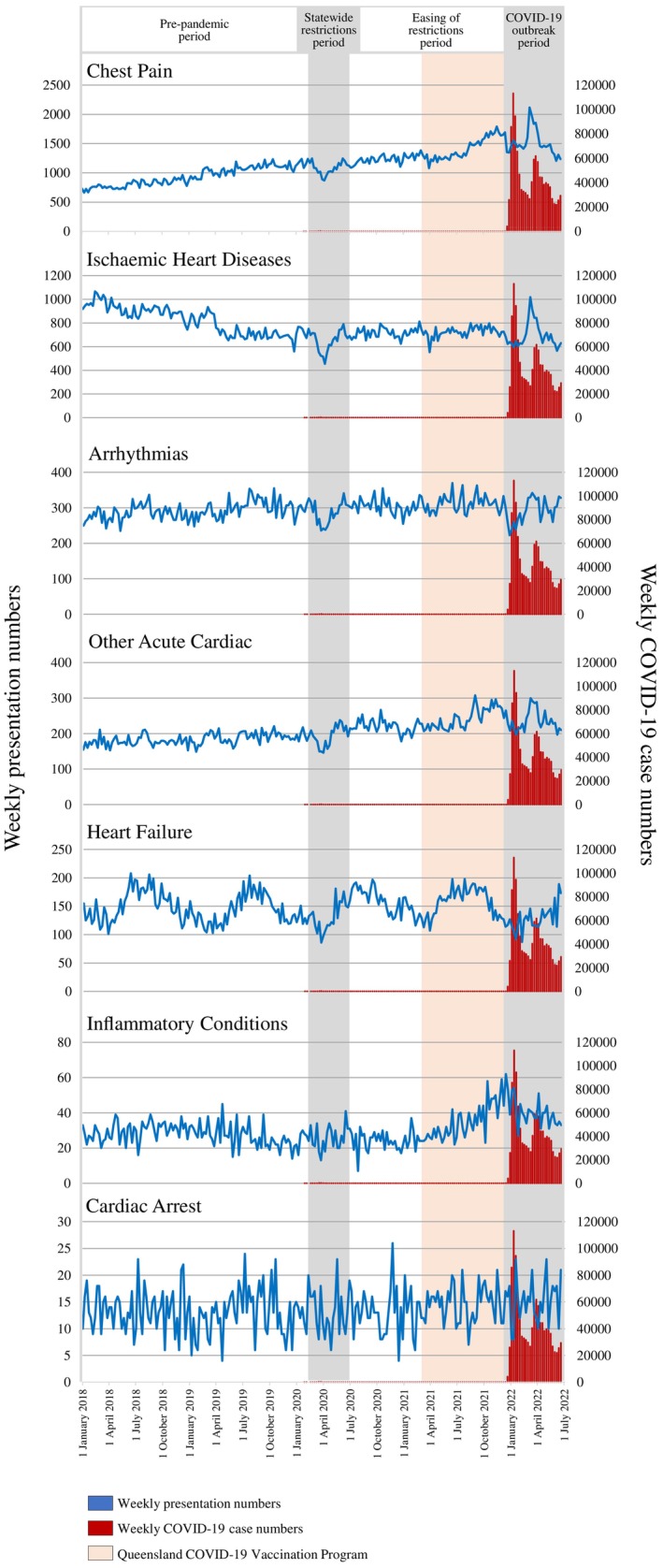
Weekly presentation numbers for acute cardiac conditions.

A noticeable decrease in weekly presentation numbers occurred shortly after the start of ‘statewide restrictions’ in Queensland for four of the seven conditions: chest pain, ischaemic heart disease, arrhythmias, and ‘other’ acute cardiac conditions (Figure 2). Acute cardiac presentations reached their lowest weekly numbers during the first two weeks of April 2020. There was a similar decrease in presentations during the first wave of COVID‐19 for the same four conditions, as well as inflammatory conditions. While heart failure visits declined during ‘statewide restrictions’ and ‘outbreak’ periods, this appeared to follow seasonality.

## 4 Discussion

During the COVID‐19 pandemic, ED presentations for acute cardiac conditions changed in varying ways in Queensland, with a small decrease in overall presentations during the initial ‘statewide restrictions’ and a subsequent larger increase during the ‘easing of restrictions’ and ‘outbreak’ periods. Fears of contracting COVID‐19 and/or overburdening healthcare settings have been reported globally as significant contributors to hospital and healthcare avoidance during the pandemic [21, 22]. However, Queensland had very few COVID‐19 cases until 2022 [12]. Social isolation caused by restrictions and social distancing measures may have limited support systems and reduced the ability for some people to access emergency care. In addition, people may have ignored symptoms or chosen to manage their symptoms at home, leading to delayed ED presentations. The, albeit small, increased proportion of ambulance presentations, out of hours arrivals, and the increase in triage category 2 allocations also suggests there is some possibility that patients may have avoided or delayed seeking care until their condition became worse, requiring more urgent care once they presented to ED, although caution in attributing clinical meaningfulness is required. Following restrictions, presentation numbers, arrival mode and arrival time returned to and exceeded pre‐pandemic levels.

Weekly presentation numbers revealed that the biggest decline in cardiac‐related ED presentations occurred during April 2020 in the first 2–4 weeks following the introduction of stay‐at‐home orders in Queensland [23]. This is similar to observations in New South Wales [24], Victoria [25] and locations overseas such as the United States [7] and Italy [2]. Public health messaging during the same time advised people to stay home if they were unwell [26]. This suggests that in Queensland, the restrictions likely played a role in the decrease in acute cardiac‐related ED presentations, similar to the impact seen for all‐cause ED presentations in Queensland [5].

During ‘easing of restrictions’, ED presentations for chest pain and inflammatory conditions increased. During this time, the Queensland COVID‐19 vaccination program was implemented with 80% of eligible people receiving the vaccine between February and December 2021 [12]. After COVID‐19 vaccination programs commenced around the world, reports indicated a small increase in pericarditis cases, particularly among younger males and after the second dose of the vaccine [27]. While our data did not include vaccination status, other possible reasons for increased presentation rates of chest pain and inflammatory conditions include heightened awareness of post‐vaccine side effects, as well as increased awareness by clinicians to look for potential pericarditis cases during the second half of 2021.

During the study period, Queensland experienced population growth [13], which may have subsequently increased overall healthcare demand. In our study, presentations for acute cardiac conditions rose by 20% (from 347 to 416 presentations per day), a notably higher rate compared to the overall Queensland population increase of 7% in the same timeframe [13]. This suggests that cardiac‐related presentations increased at a rate faster than population growth, aligning with current increases in all‐cause ED presentations across Australian EDs [28]. The rise in ischaemic heart disease and chest pain presentations after the first wave of COVID‐19 in 2022 may be a manifestation of the broader impact of SARS‐CoV‐2 infection and associated (pandemic) behaviour on cardiovascular health.

The observed changes in acute cardiac‐related ED presentations in Queensland are similar to other Australian states where ED presentations declined despite low COVID‐19 case numbers [4, 24, 29]. While this study did not investigate causes for the changes, it is possible that there were variations in health‐seeking behaviour during periods of pandemic restrictions, rather than changes in the prevalence or incidence of cardiac disease or COVID‐19. These behavioural changes may have been driven by factors such as adherence to government restrictions, fear of contracting COVID‐19 in healthcare settings, confusion about when to seek medical care, or a perceived need to avoid burdening the healthcare system.

## 5 Limitations

This was an analysis of routinely collected data from a large database sourced from across all public EDs in Queensland. Despite this, there are four main limitations of this study. First, data used for this study were limited to that available within a minimum data set. Whilst this provided a breadth of information, data were limited in depth. Future research might consider data linkage to provide an understanding of the patient journey before and after the ED episode of care, and/or incorporate Patient Reported Experience Measures (PREMs) and/or Patient Reported Outcome Measures (PROMs). Second, our findings are reflective of presentations made to public EDs only. Whilst most hospital EDs in Queensland are public, many private hospitals provide specialist cardiac services and adjusted service delivery during the pandemic. Thus, the impact on private EDs warrants consideration in future research. Third, potential variations in the way clinicians record ED diagnoses could lead to slightly different ICD‐10‐AM codes between hospital and health service areas. The ICD‐10‐AM codes used in our study were drawn from previous literature and in consultation with expert clinician researchers, and categorised to reduce potential variations. Fourth, the retrospective nature of this study means there may have been other potential confounding factors that were not incorporated. Statistically significant changes may not translate to being clinically meaningful in the practice setting of the ED. The study highlights changes in cardiac‐related ED presentations during the pandemic but does not assess whether this has had any impact on patient morbidity and/or mortality. A prospective longitudinal study tracking clinical outcomes would be a valuable extension to this research. Furthermore, the study design (retrospective cohort) does not enable us to determine any cause‐and‐effect relationship.

## 6 Conclusion

The COVID‐19 pandemic impacted acute cardiac‐related ED presentations in Queensland in various ways. Incorporating clear and consistent guidance into public health messaging is crucial during pandemics, particularly emphasising the need for individuals to seek timely medical care for urgent cardiac symptoms, such as chest pain, shortness of breath, or other warning signs of serious conditions. Ensuring people understand when and how to access emergency care, even during times of restrictions, is vital to prevent delays in treatment and reduce the risk of adverse health outcomes. Furthermore, public health messaging should reassure the public that EDs remain available for use during future pandemics.

## Author Contributions

**Emma J. Hall:** conceptualisation, methodology, data curation, analysis, writing – original draft, visualisation. **Gerben Keijzers:** conceptualisation, methodology, writing – review and editing, supervision. **Jamie Ranse:** conceptualisation, methodology, writing – review and editing, supervision. **Amy L. Sweeny:** methodology, data curation, analysis, writing – review and editing. **Julia Crilly:** conceptualisation, methodology, writing – review and editing, supervision.

## Ethics Statement

Ethics approvals were received from the Gold Coast Hospital and Health Service (LNR/2020/QGC/65436) and Griffith University (2022/766) Human Research Ethics Committees. Approval for the use of EDC data was granted as per Public Health Act 2005 (Qld) requirements.

## Conflicts of Interest

The views and findings presented in this paper are those of the investigators and do not represent those of the collaborating organisations. Author Gerben Keijzers is an Editorial Board member of EMA. He had no involvement in editorial decisions relating to this manuscript. COVERED‐COVID Study Investigator Jaimi Greenslade is an Editorial Board member of EMA and had no involvement in editorial decisions relating to this manuscript.

## Supporting information


**Data S1.** Supporting Information.

## Data Availability

We are unable to share or make publicly available data used for this study due to ethical and data privacy requirements.
