# Supplementary material for: The Impact of the COVID‐19 Pandemic on Cardiac Related Emergency Department Presentations in Queensland: A Retrospective Cohort Study
Source: Emerg Med Australas. 2025 Jul 24;37(4):e70096. doi: 10.1111/1742-6723.70096 (PMC12290241; doi:10.1111/1742-6723.70096)
Supplement: Supplementary file 1 — Data S1. Supporting Information. [file EMM-37-0-s001.docx]

**Table S1. ICD-10-AM categorisation of cardiac conditions used in this study**

| **ICD-10-AM and DESCRIPTION** | **AIHW SUBCATEGORY** | **AIHW CATEGORY** | **RECODED CATEGORIES FOR ANALYSIS** |
| --- | --- | --- | --- |
| I20.0 Unstable angina | Angina pectoris | Ischaemic heart diseases | IHD |
| I21.9 Acute myocardial infarction, unspecified | Acute myocardial infarction | Ischaemic heart diseases | IHD |
| I21.4 Acute subendocardial myocardial infarction (NSTEMI) | Acute myocardial infarction | Ischaemic heart diseases | IHD |
| I24.8 Other forms of acute ischaemic heart disease | Other acute ischaemic heart diseases | Ischaemic heart diseases | IHD |
| I21.3 Acute transmural myocardial infarction of unspecified site (STEMI) | Acute myocardial infarction | Ischaemic heart diseases | IHD |
| I20.9 Angina pectoris, unspecified | Angina pectoris | Ischaemic heart diseases | IHD |
| I21 Acute myocardial infarction | Acute myocardial infarction | Ischaemic heart diseases | IHD |
| I24.9 Acute ischaemic heart disease, unspecified | Other acute ischaemic heart diseases | Ischaemic heart diseases | IHD |
| I21.0 Acute transmural myocardial infarction of anterior wall | Acute myocardial infarction | Ischaemic heart diseases | IHD |
| I21.1 Acute transmural myocardial infarction of inferior wall | Acute myocardial infarction | Ischaemic heart diseases | IHD |
| I20 Angina pectoris | Angina pectoris | Ischaemic heart diseases | IHD |
| I20.1 Angina pectoris with documented spasm | Angina pectoris | Ischaemic heart diseases | IHD |
| I20.8 Other forms of angina pectoris | Angina pectoris | Ischaemic heart diseases | IHD |
| I21.2 Acute transmural myocardial infarction of other sites | Acute myocardial infarction | Ischaemic heart diseases | IHD |
| I22 Subsequent myocardial infarction | Subsequent myocardial infarction | Ischaemic heart diseases | IHD |
| I22.0 Subsequent myocardial infarction of anterior wall | Subsequent myocardial infarction | Ischaemic heart diseases | IHD |
| I22.1 Subsequent myocardial infarction of inferior wall | Subsequent myocardial infarction | Ischaemic heart diseases | IHD |
| I22.8 Subsequent myocardial infarction of other sites | Subsequent myocardial infarction | Ischaemic heart diseases | IHD |
| I22.9 Subsequent myocardial infarction of unspecified site | Subsequent myocardial infarction | Ischaemic heart diseases | IHD |
| I24.0 Coronary thrombosis not resulting in myocardial infarction | Other acute ischaemic heart diseases | Ischaemic heart diseases | IHD |
| I24.1 Dressler's syndrome | Other acute ischaemic heart diseases | Ischaemic heart diseases | IHD |
| I30.9 Acute pericarditis, unspecified | Acute pericarditis | Other forms of heart disease | INFLAMMATORY |
| I33.0 Acute and subacute infective endocarditis | Acute and subacute endocarditis | Other forms of heart disease | INFLAMMATORY |
| I38 Endocarditis, valve unspecified | Endocarditis, valve unspecified | Other forms of heart disease | INFLAMMATORY |
| I40.9 Acute myocarditis, unspecified | Acute myocarditis | Other forms of heart disease | INFLAMMATORY |
| I30 Acute pericarditis | Acute pericarditis | Other forms of heart disease | INFLAMMATORY |
| I30.0 Acute nonspecific idiopathic pericarditis | Acute pericarditis | Other forms of heart disease | INFLAMMATORY |
| I30.1 Infective pericarditis | Acute pericarditis | Other forms of heart disease | INFLAMMATORY |
| I30.8 Other forms of acute pericarditis | Acute pericarditis | Other forms of heart disease | INFLAMMATORY |
| I33 Acute and subacute endocarditis | Acute and subacute endocarditis | Other forms of heart disease | INFLAMMATORY |
| I33.9 Acute endocarditis, unspecified | Acute and subacute endocarditis | Other forms of heart disease | INFLAMMATORY |
| I39 Endocarditis and heart valve disorders in diseases classified elsewhere | Endocarditis and heart valve disorders in diseases classified elsewhere | Other forms of heart disease | INFLAMMATORY |
| I40 Acute myocarditis | Acute myocarditis | Other forms of heart disease | INFLAMMATORY |
| I40.0 Infective myocarditis | Acute myocarditis | Other forms of heart disease | INFLAMMATORY |
| I40.1 Isolated myocarditis | Acute myocarditis | Other forms of heart disease | INFLAMMATORY |
| I40.8 Other acute myocarditis | Acute myocarditis | Other forms of heart disease | INFLAMMATORY |
| I41 Myocarditis in diseases classified elsewhere | Myocarditis in diseases classified elsewhere | Other forms of heart disease | INFLAMMATORY |
| I51.4 Myocarditis, unspecified | Complications and ill-defined descriptions of heart disease | Other forms of heart disease | INFLAMMATORY |
| I48 Atrial fibrillation and flutter | Atrial fibrillation and flutter | Other forms of heart disease | ARRHYTHMIAS |
| I48.9 Atrial fibrillation and atrial flutter, unspecified | Atrial fibrillation and flutter | Other forms of heart disease | ARRHYTHMIAS |
| I47.1 Supraventricular tachycardia | Paroxysmal tachycardia | Other forms of heart disease | ARRHYTHMIAS |
| I49.9 Cardiac arrhythmia, unspecified | Other cardiac arrhythmias | Other forms of heart disease | ARRHYTHMIAS |
| I44.2 Atrioventricular block, complete | Atrioventricular and left bundle-branch block | Other forms of heart disease | ARRHYTHMIAS |
| I47.2 Ventricular tachycardia | Paroxysmal tachycardia | Other forms of heart disease | ARRHYTHMIAS |
| I48.0 Paroxysmal atrial fibrillation | Atrial fibrillation and flutter | Other forms of heart disease | ARRHYTHMIAS |
| I45.6 Pre-excitation syndrome | Other conduction disorders | Other forms of heart disease | ARRHYTHMIAS |
| I45.9 Conduction disorder, unspecified | Other conduction disorders | Other forms of heart disease | ARRHYTHMIAS |
| I49.3 Ventricular premature depolarisation | Other cardiac arrhythmias | Other forms of heart disease | ARRHYTHMIAS |
| I49.5 Sick sinus syndrome | Other cardiac arrhythmias | Other forms of heart disease | ARRHYTHMIAS |
| I49.8 Other specified cardiac arrhythmias | Other cardiac arrhythmias | Other forms of heart disease | ARRHYTHMIAS |
| I44 Atrioventricular and left bundle-branch block | Atrioventricular and left bundle-branch block | Other forms of heart disease | ARRHYTHMIAS |
| I44.0 Atrioventricular block, first degree | Atrioventricular and left bundle-branch block | Other forms of heart disease | ARRHYTHMIAS |
| I44.1 Atrioventricular block, second degree | Atrioventricular and left bundle-branch block | Other forms of heart disease | ARRHYTHMIAS |
| I44.3 Other and unspecified atrioventricular block | Atrioventricular and left bundle-branch block | Other forms of heart disease | ARRHYTHMIAS |
| I44.4 Left anterior fascicular block | Atrioventricular and left bundle-branch block | Other forms of heart disease | ARRHYTHMIAS |
| I44.7 Left bundle-branch block, unspecified | Atrioventricular and left bundle-branch block | Other forms of heart disease | ARRHYTHMIAS |
| I45 Other conduction disorders | Other conduction disorders | Other forms of heart disease | ARRHYTHMIAS |
| I45.1 Other and unspecified right bundle-branch block | Other conduction disorders | Other forms of heart disease | ARRHYTHMIAS |
| I45.2 Bifascicular block | Other conduction disorders | Other forms of heart disease | ARRHYTHMIAS |
| I45.3 Trifascicular block | Other conduction disorders | Other forms of heart disease | ARRHYTHMIAS |
| I45.5 Other specified heart block | Other conduction disorders | Other forms of heart disease | ARRHYTHMIAS |
| I45.8 Other specified conduction disorders | Other conduction disorders | Other forms of heart disease | ARRHYTHMIAS |
| I47 Paroxysmal tachycardia | Paroxysmal tachycardia | Other forms of heart disease | ARRHYTHMIAS |
| I48.1 Persistent atrial fibrillation | Atrial fibrillation and flutter | Other forms of heart disease | ARRHYTHMIAS |
| I48.2 Chronic atrial fibrillation | Atrial fibrillation and flutter | Other forms of heart disease | ARRHYTHMIAS |
| I48.3 Typical atrial flutter | Atrial fibrillation and flutter | Other forms of heart disease | ARRHYTHMIAS |
| I48.4 Atypical atrial flutter | Atrial fibrillation and flutter | Other forms of heart disease | ARRHYTHMIAS |
| I49 Other cardiac arrhythmias | Other cardiac arrhythmias | Other forms of heart disease | ARRHYTHMIAS |
| I49.0 Ventricular fibrillation and flutter | Other cardiac arrhythmias | Other forms of heart disease | ARRHYTHMIAS |
| I49.1 Atrial premature depolarisation | Other cardiac arrhythmias | Other forms of heart disease | ARRHYTHMIAS |
| I49.2 Junctional premature depolarisation | Other cardiac arrhythmias | Other forms of heart disease | ARRHYTHMIAS |
| I49.4 Other and unspecified premature depolarisation | Other cardiac arrhythmias | Other forms of heart disease | ARRHYTHMIAS |
| I46.9 Cardiac arrest, unspecified | Cardiac arrest | Other forms of heart disease | CARDIAC ARREST |
| I46 Cardiac arrest | Cardiac arrest | Other forms of heart disease | CARDIAC ARREST |
| I46.0 Cardiac arrest with successful resuscitation | Cardiac arrest | Other forms of heart disease | CARDIAC ARREST |
| I46.1 Sudden cardiac death, so described | Cardiac arrest | Other forms of heart disease | CARDIAC ARREST |
| I50 Heart failure | Heart failure | Other forms of heart disease | HF/CARDIOMYOPATHY |
| I50.9 Heart failure, unspecified | Heart failure | Other forms of heart disease | HF/CARDIOMYOPATHY |
| I50.1 Left ventricular failure | Heart failure | Other forms of heart disease | HF/CARDIOMYOPATHY |
| I42.9 Cardiomyopathy, unspecified | Cardiomyopathy | Other forms of heart disease | HF/CARDIOMYOPATHY |
| I42 Cardiomyopathy | Cardiomyopathy | Other forms of heart disease | HF/CARDIOMYOPATHY |
| I42.0 Dilated cardiomyopathy | Cardiomyopathy | Other forms of heart disease | HF/CARDIOMYOPATHY |
| I42.1 Obstructive hypertrophic cardiomyopathy | Cardiomyopathy | Other forms of heart disease | HF/CARDIOMYOPATHY |
| I42.2 Other hypertrophic cardiomyopathy | Cardiomyopathy | Other forms of heart disease | HF/CARDIOMYOPATHY |
| I42.3 Endomyocardial (eosinophilic) disease | Cardiomyopathy | Other forms of heart disease | HF/CARDIOMYOPATHY |
| I42.4 Endocardial fibroelastosis | Cardiomyopathy | Other forms of heart disease | HF/CARDIOMYOPATHY |
| I42.5 Other restrictive cardiomyopathy | Cardiomyopathy | Other forms of heart disease | HF/CARDIOMYOPATHY |
| I42.6 Alcoholic cardiomyopathy | Cardiomyopathy | Other forms of heart disease | HF/CARDIOMYOPATHY |
| I42.7 Cardiomyopathy due to drugs and other external agents | Cardiomyopathy | Other forms of heart disease | HF/CARDIOMYOPATHY |
| I42.8 Other cardiomyopathies | Cardiomyopathy | Other forms of heart disease | HF/CARDIOMYOPATHY |
| I43 Cardiomyopathy in diseases classified elsewhere | Cardiomyopathy in diseases classified elsewhere | Other forms of heart disease | HF/CARDIOMYOPATHY |
| I43.0 Cardiomyopathy in infectious and parasitic diseases classified elsewhere | Cardiomyopathy in diseases classified elsewhere | Other forms of heart disease | HF/CARDIOMYOPATHY |
| I43.1 Cardiomyopathy in metabolic diseases | Cardiomyopathy in diseases classified elsewhere | Other forms of heart disease | HF/CARDIOMYOPATHY |
| I43.8 Cardiomyopathy in other diseases classified elsewhere | Cardiomyopathy in diseases classified elsewhere | Other forms of heart disease | HF/CARDIOMYOPATHY |
| I50.0 Congestive heart failure | Heart failure | Other forms of heart disease | HF/CARDIOMYOPATHY |
| R07.4 Chest pain, unspecified | Pain in throat and chest | Symptoms and signs involving the circulatory and respiratory systems | CHEST PAIN |
| R07.3 Other chest pain | Pain in throat and chest | Symptoms and signs involving the circulatory and respiratory systems | CHEST PAIN |
| R00.2 Palpitations | Abnormalities of heart beat | Symptoms and signs involving the circulatory and respiratory systems | OTHER |
| R00.1 Bradycardia, unspecified | Abnormalities of heart beat | Symptoms and signs involving the circulatory and respiratory systems | OTHER |
| R00.0 Tachycardia, unspecified | Abnormalities of heart beat | Symptoms and signs involving the circulatory and respiratory systems | OTHER |
| I01 Rheumatic fever with heart involvement | Rheumatic fever with heart involvement | Acute rheumatic fever | OTHER |
| I01.0 Acute rheumatic pericarditis | Rheumatic fever with heart involvement | Acute rheumatic fever | OTHER |
| I01.1 Acute rheumatic endocarditis | Rheumatic fever with heart involvement | Acute rheumatic fever | OTHER |
| I01.2 Acute rheumatic myocarditis | Rheumatic fever with heart involvement | Acute rheumatic fever | OTHER |
| I01.8 Other acute rheumatic heart disease | Rheumatic fever with heart involvement | Acute rheumatic fever | OTHER |
| I01.9 Acute rheumatic heart disease, unspecified | Rheumatic fever with heart involvement | Acute rheumatic fever | OTHER |
| I23 Certain current complications following acute myocardial infarction | Certain current complications following acute myocardial infarction | Ischaemic heart diseases | OTHER |
| I23.1 Atrial septal defect as current complication following acute myocardial infarction | Certain current complications following acute myocardial infarction | Ischaemic heart diseases | OTHER |
| I23.2 Ventricular septal defect as current complication following acute myocardial infarction | Certain current complications following acute myocardial infarction | Ischaemic heart diseases | OTHER |
| I23.6 Thrombosis of atrium, auricular appendage, and ventricle as current complications following acute myocardial infarction | Certain current complications following acute myocardial infarction | Ischaemic heart diseases | OTHER |
| I23.8 Other current complications following acute myocardial infarction | Certain current complications following acute myocardial infarction | Ischaemic heart diseases | OTHER |
| I24 Other acute ischaemic heart diseases | Other acute ischaemic heart diseases | Ischaemic heart diseases | OTHER |
| I35.0 Aortic (valve) stenosis | Nonrheumatic aortic valve disorders | Other forms of heart disease | OTHER |
| I51.3 Intracardiac thrombosis, not elsewhere classified | Complications and ill-defined descriptions of heart disease | Other forms of heart disease | OTHER |
| AIHW: Australian Institute of Health and Welfare; ICD-10-AM: International Statistical Classification of Diseases, tenth revision, Australian modification; IHD: Ischaemic Heart Disease; HF: Heart Failure; NSTEMI: Non-ST segment elevation myocardial infarction; STEMI: ST-elevation myocardial infarction  AIHW categories accessed 19 April 2023 from: https://www.ihacpa.gov.au/health-care/classification/emergency-care/emergency-department-icd-10-am-principal-diagnosis-short-list | | | |
|  |  |  |  |

**Table S2. Data cleaning and data coding of variables used in this study**

| **Variable** | **SPSS Recoding from Emergency Data Collection** |
| --- | --- |
| Sex | RECODE fgender (1=1) (2=2) (3=SYSMIS) (9=SYSMIS) INTO r_Sex. |
| Age category (years) | RECODE PAT_AGE (Lowest thru 24=1) (25 thru 34=2) (35 thru 44=3) (45 thru 54=4) (55 thru 64=5) (65 thru 74=6) (75 thru 84=7) (85 thru Highest=8) INTO r_AgeCat.  *age >105 years removed |
| Aboriginal and Torres Strait Islander origin | RECODE fATSI ('Aboriginal but not Torres Strait Islander origin'=1) ('Torres Strait Islander but '+ 'not Aboriginal origin'=1) ('Both Aboriginal and Torres Strait Islander origin'=1) ('Neither '+ 'Aboriginal nor Torres Strait Islander origin'=2) ('Not stated or unknown'=3) INTO r_ATSI. |
| SEIFA (Socio-Economic Indexes for Areas) | Postcode used to allocate SEIFA as per Australia Bureau of Statistics: Socio-Economic Indexes for Areas (SEIFA), Australia. 2021. Available from: https://www.abs.gov.au/statistics/people/people-and-communities/socio-economic-indexes-areas-seifa-australia/2021#index-of-relative-socio-economic-disadvantage-irsd- |
| Remoteness (Residential postcode) | Postcode used to allocate remoteness as per Australia Bureau of Statistics: Australian Statistical Geography Standard (ASGS): Volume 5 - Remoteness Structure, July 2016 correspondence, 2017 Postcode to 2016 Remoteness Area. 2016. Available from: https://www.abs.gov.au/AUSSTATS/abs@.nsf/DetailsPage/1270.0.55.005July%202016?OpenDocument |
| Residential (Geographical) Location | RECODE r_Postcode_State ('QLD'='QLD') ('NSW'='Interstate') ('NT'='Interstate') ('SA'='Interstate') ('TAS'='Interstate') ('VIC'='Interstate') ('WA'='Interstate') ('Overseas'='Overseas') ('No fixed '+ 'address'='SYSMIS') ('Unknown'='SYSMIS') ('ACT'='Interstate') INTO r_State_QLDorOther. |
| Day of Arrival | RECODE r_DayOfWeek ('MON'=1) ('TUE'=1) ('WED'=1) ('THU'=1) ('FRI'=1) ('SAT'=2) ('SUN'=2) INTO r_WeekdayWeekend |
| Time of Arrival | Time of Arrival first converted from ‘time’ format to ‘number’ format  RECODE r_AmPm_for_recode (21600 thru 64740=1) (0 thru 21540=2) (64800 thru 86340=2) INTO r_AmPm.  (Am = in-hours 06:00-17:59, Pm = out-of-hours 18:00-05:59) |
| Mode of Arrival | RECODE farrivalmode ('Ambulance (fixed wing aircraft) '='ambulance') ('Ambulance '+ '(helicopter)'='ambulance') ('Ambulance (road - paramedic)'='ambulance') ('Ambulance (road - '+ 'patient transport officer)'='ambulance') ('Other'='other') ('Police or Correctional Services '+ 'vehicle'='police') ('Walked in / public or private transport'='walk in') ('Community services '+ 'vehicle'='walk in') INTO r_ArrivalMode. |
| End Status | RECODE EPISODE_END_STATUS ('Admitted'='Admit') ('Admitted to Hospital in the Home service'='Admit') ('Admitted to observation ward'='Admit') ('Admitted to Short Stay Unit'='Short Stay Unit') ('Admitted to the emergency department / service'='Admit') ('Died in the emergency department / '+ 'service'='Died in ED') ('Emergency service episode completed and discharged'='Home') ('Hospital in the Home patient transferred to ward'='Admit') ('Returned to Hospital in the Home '+ 'service'='Admit') ('Transferred to another hospital'='Transfer') ('Did not wait'='Did Not '+ 'Wait/LAMA') ('Left at own risk after treatment commenced'='Did Not Wait/LAMA') INTO r_endstatus. |
| ED Length of Stay | COMPUTE EDLOS_min=DATEDIFF(PHYSICAL_DEPART_DATETIME, PRESENTATION_DATETIME, "minutes")  •RECODE EDLOS_min (0 thru 1323=Copy) (ELSE=SYSMIS) INTO r_LOSclean.  * negative values removed.  * top 0.5% of presentations removed (everything >1323 min).  •RECODE r_LOSclean (Lowest thru 239=1) (240 thru Highest=2) INTO LOS_4hr. |
